# The Nitrogen Atom of Vitamin B_6_ Is Essential for the Catalysis of Radical Aminomutases

**DOI:** 10.3390/ijms23095210

**Published:** 2022-05-06

**Authors:** Amarendra Nath Maity, Jun-Ru Chen, Quan-Yuan Li, Shyue-Chu Ke

**Affiliations:** Physics Department, National Dong Hwa University, Hualien 974301, Taiwan; anmaity@gmail.com (A.N.M.); 410114237@gms.ndhu.edu.tw (J.-R.C.); 610614013@gms.ndhu.edu.tw (Q.-Y.L.)

**Keywords:** PLP, dAdoCbl, coenzyme B_12_, aminomutase, mutagenesis, EPR, DFT

## Abstract

Radical aminomutases are pyridoxal 5′-phosphate (PLP, a B_6_ vitamer)-dependent enzymes that require the generation of a 5′-deoxyadenosyl radical to initiate the catalytic cycle, to perform a 1,2 amino group shift reaction. The role of the nitrogen atom of PLP in radical aminomutases has not been investigated extensively yet. We report an alternative synthetic procedure to provide easy access to 1-deazaPLP (dAPLP), an isosteric analog of PLP which acts as a probe for studying the role of the nitrogen atom. Our results revealed that lysine 5,6-aminomutase (5,6-LAM), a radical aminomutase, reconstituted with dAPLP cannot turn over a substrate, demonstrating that the nitrogen atom is essential for radical aminomutases. In contrast, biochemical and spectroscopic studies on the S238A variant reconstituted with PLP revealed a minuscule loss of activity. This apparent anomaly can be explained by a water-mediated rescue of activity in S238A, as if mimicking the active site of lysine 2,3-aminomutase. This study leads to a better comprehension of how enzymes harness the optimum capability of PLP to realize catalysis.

## 1. Introduction

**Figure 1:**
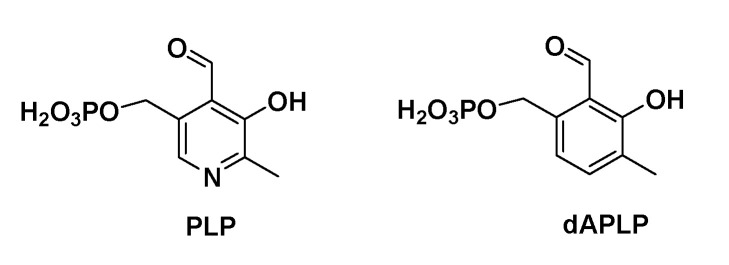
Chemical structures of PLP and 1-deaza-PLP (dAPLP).

Vitamin B_6_ plays many physiological roles [1]. Pyridoxal 5′-phosphate (PLP, Figure 1) is the biologically active form of vitamin B_6_ [2]. A vast number of enzymes employ the catalytic repertoire of PLP as a cofactor to accomplish a range of challenging chemical transformations, including transamination, racemization, and elimination reactions [3,4,5,6]. Moreover, the versatility of PLP has inspired the application of it in various fields such as biomimetic catalysis [7,8], biomolecule labeling [9], combinatorial biosynthesis [10], and biomimetic chirality sensing [11].

**Scheme 1:**
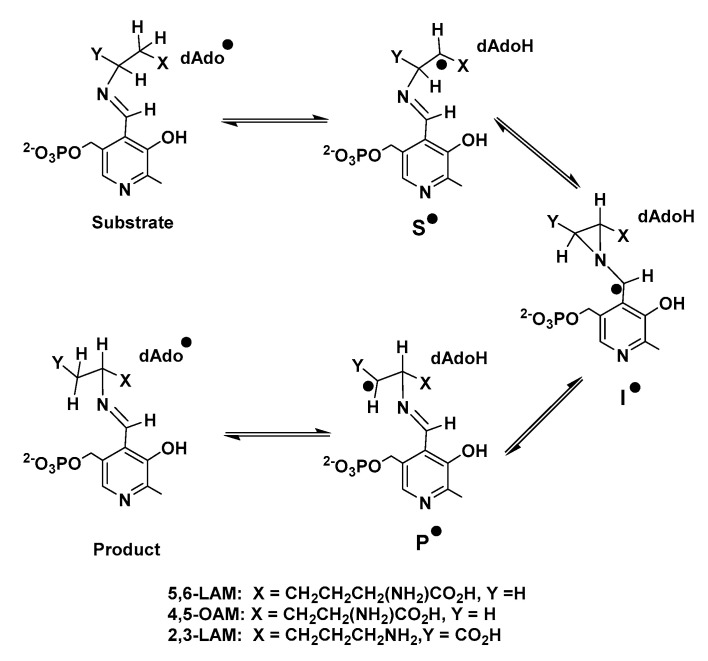
Proposed reaction mechanism of radical aminomutases.

PLP-dependent enzymes (henceforth referred to as PLPoenzymes following the analogy of metalloenzyme and flavoenzyme) can be classified into five different families depending upon their respective folds. Aspartate aminotransferase (AAT), tryptophan synthase, alanine racemase (AR), D-amino acid aminotransferase, and glycogen phosphorylase represent enzymes of fold types I, II, III, IV, and V, respectively [12]. There is another type of PLPoenzymes which cannot be placed into any of these five families. Radical aminomutases such as lysine 5,6-aminomutase (5,6-LAM), ornithine 4,5-aminomutase (4,5-OAM), lysine 2,3-aminomutase (2,3-LAM), and glutamate 2,3-aminomutase (2,3-GAM), belong to this family [13,14]. The generation of a 5′-deoxyadenosyl radical (dAdo^•^) is required in these dAdo- and PLP-codependent radical enzymes, which follow a similar reaction mechanism (Scheme 1) involving three PLP-bound radical intermediates—a substrate related radical (S^•^), an intermediate azacyclopropylcarbinyl radical (I^•^), and a product-related radical (P^•^)—to catalyze the reaction of the 1,2 amino group shift [13,15,16]. Nevertheless, two different modes of generation of dAdo^•^ are observed among radical aminomutases. 5,6-LAM and 4,5-OAM [17] use coenzyme B_12_ (5′-deoxyadenosylcobalamin, dAdoCbl) as the source of dAdo^•^, while 2,3-LAM and 2,3-GAM [14] use S-adenosylmethionine (AdoMet or SAM) [18,19,20]. Among these enzymes 2,3-LAM has been studied most extensively, and the corresponding S^•^ and P^•^ have already been identified in the case of 2,3-LAM by Frey and coworkers [13,20,21]. dAdo^•^ was very recently detected and characterized in SAM-dependent pyruvate formate-lyase activating enzyme (PFL-AE) by Yang and coworkers [22]. Later, Sayler and coworkers also captured dAdo^•^ in another SAM-dependent radical enzyme, HydG, with a non-native substrate [23].

5,6-LAM shares features of fold types II, III, and IV of PLPoenzymes. The similarity to fold type II is the fact that a serine residue (Ser238α in 5,6-LAM) interacts with the pyridine nitrogen of PLP through a hydrogen bond [24]. 5,6-LAM, a bacterial enzyme, is known to have some interesting features: it can accept at least three substrates—D-lysine, L-β-lysine, and L-lysine [25]; it undergoes substrate-dependent suicide inactivation [26]; it undergoes large scale conformational movement to accomplish catalysis by effecting the reversible transition between the resting open state and the closed state [27]; and it shows a magnetic field effect [28]. Several aspects of the mechanism of action have been established using electron paramagnetic resonance (EPR) and electron nuclear double resonance (ENDOR) spectroscopies, synthetic isotopologues, and density functional theoretical (DFT) computations [29]. The use of synthetic, site-directed, labelled substrate analogues [30,31] have unambiguously characterized the corresponding S^•^ in the reaction of 5,6-LAM [32]. Substrate homologues and analogues, along with site-directed mutagenesis, shed light on the binding of the substrate and the release of the product [33,34]. Recently, a noncovalent transfer of spin density from the substrate-related radical to the N7 of deoxyadenosine as one of the facilitators for the catalysis in 5,6-LAM was unraveled using ^15^N-labelled 5,6-LAM and electron-spin-echo envelope modulation (ESEEM) spectroscopy [35].

5,6-LAM is an α_2_β_2_ heterodimer and is homologous to 4,5-OAM. Despite many similarities, there are a few differences. The most striking difference is that 4,5-OAM is substrate-specific to D-ornithine, whereas 5,6-LAM, being substrate tolerant, can turn over three isomers of lysine. Secondly, 5,6-LAM undergoes suicide inactivation ten times faster than 4,5-OAM. The chemical structure of PLP comprises a pyridine ring containing several functional groups: an aldehyde, a phenolic hydroxyl, a methylene phosphate moiety, and a methyl group. These groups play various important roles in binding as well as catalysis. The pyridine ring is believed to function as an electron sink during catalysis. Nevertheless, a phenyl ring should also function similarly to a pyridine ring. The presence of the nitrogen atom ensues three different possible scenarios in the active sites of various PLPoenzymes: first, protonated pyridine nitrogen interacting with an aspartate residue through salt bridge interaction, as in fold type I; and second, pyridine nitrogen interacting through hydrogen bonding either with a serine residue, as in fold type II, or an arginine residue, as in fold type III. Among the radical aminomutases, conserved serine residues are present to form a hydrogen bond with pyridine nitrogen in the active sites of 5,6-LAM and 4,5-OAM, as in fold type II PLPoenzymes, whereas in the case of 2,3-LAM, a water molecule forms a hydrogen bond with the pyridine nitrogen of PLP [36]. Depending upon the requirement of the reaction involved, these interactions are not only helpful for the binding of PLP to protein, but also as a facilitator for catalysis. In the third scenario, formally unprotonated pyridine nitrogen is believed to be present in the mutants while it faces alanine in its close vicinity. The roles of these interactions have traditionally been studied by employing site-directed mutagenesis of these conserved residues [37,38,39,40,41]. Aspartate residue is the most common residue to interact with pyridine nitrogen atoms in PLPoenzymes such as AAT. This interaction is crucial for stabilizing an important reaction intermediate, which is known as quinonoid intermediate. Mutation of this conserved aspartate to alanine leads to a loss of activity of AAT, as the quinonoid intermediate cannot be stabilized in the mutant due to the absence of the carboxylate moiety that interacts with protonated pyridine nitrogen [5]. In the case of AR, the interaction between arginine and pyridine nitrogen was explored through various mutations of this residue [37]. Mutations of O-acetylserine sulfhydrylase (OASS) [38] and the β-subunit of tryptophan synthase (β-TRPS) [40] revealed interesting features. Both OASS and β-TRPS have a serine residue that interacts with the nitrogen atom of PLP and perform an α,β-elimination reaction. Nevertheless, the mechanism followed by these are different. OASS follows the E_2_ mechanism while β-TRPS follows the E_1_cB mechanism [42]. PLP-N-oxide (PLP-NO, a synthetic analogue of PLP) has also provided remarkable insights in the case of AAT [43,44], tryptophanase [43,44], glycogen phosphorylase [45] and 5,6-LAM [46]. The recent synthesis of 1-deazapyridoxal 5′-phosphate (dAPLP) [47], the isosteric carbocyclic analogue of PLP (Figure 1), paved the way for studying the role of pyridine nitrogen in a more elaborate manner [48]. Griswold and Toney investigated the impact of dAPLP on three classes of PLPoenzymes represented by AAT, AR, and OASS [5]. The class of radical aminomutases was not studied in this previous report. The role of PLP and other aspects of radical aminomutases have been studied using computational methods [49,50,51], especially DFT computations [27,52,53,54,55,56].

In this paper, we present an alternative synthetic path for the easy access of dAPLP. Moreover, we report a comprehensive study involving biochemical and spectroscopic investigations on 5,6-LAM reconstituted with dAPLP, and the S238A variant of 5,6-LAM reconstituted with PLP, to decipher the role of the nitrogen atom of PLP in the catalysis of radical aminomutases. Additionally, we perform DFT and molecular modelling studies to explain those observations.

## 2. Results and Discussion

### 2.1. Synthesis of dAPLP

**Scheme 2:**
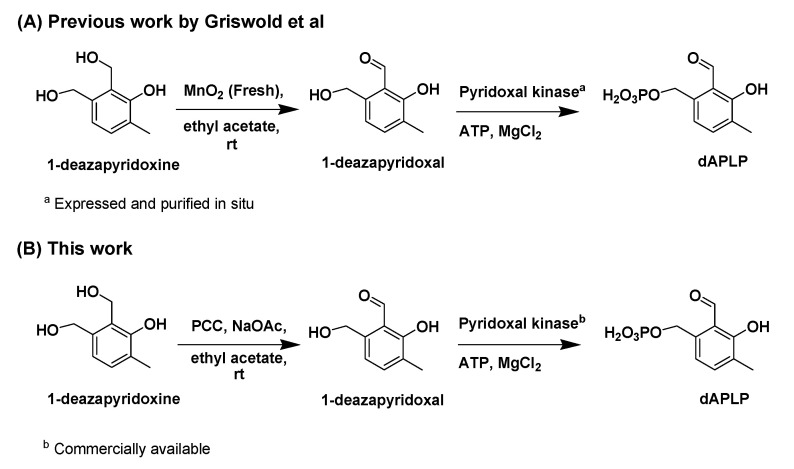
Chemoenzymatic synthesis of dAPLP. (**A**), The reaction steps followed by Griswold et al; (**B**), The reaction steps followed in this work.

The synthesis of dAPLP was performed by following the strategy reported by Griswold and co-workers [47]. In this work, we synthesized dAPLP through an alternative path to provide easy access to dAPLP (Scheme 2). The final two steps involved the oxidation of 1-deazapyridoxine to 1-deazapyridoxal, followed by the enzymatic phosphorylation of 1-deazapyridoxal to dAPLP. The penultimate step of oxidation in the scheme was achieved, in the present work, using pyridinium chlorochromate (PCC) instead of MnO_2_. The main advantage of PCC over MnO_2_ is that commercial PCC can be used as is, while freshly prepared MnO_2_ is required to achieve oxidation, as commercial MnO_2_ was ineffective. Even with freshly prepared MnO_2_, more than 25% of 1-deazapyridoxine was found to be unreacted in our case. Similarly, with PCC, 28% of 1-deazapyridoxine was unreacted. According to the previous report [47], the reaction mixture was directly used, without separation from 1-deazapyridoxine, for phosphorylation with the enzyme. We found that this unreacted 1-dezapyridoxine hampers the phosphorylation of 1-deazapyridoxal, as 1-deazapyridoxine may block the active site of the enzyme. Therefore, we recovered unreacted 1-deazapyridoxine by separating it from the mixture of oxidation products prior to enzymatic phosphorylation. The phosphorylation was performed, in the previous report, using an enzyme that was obtained through cloning, expression, and the purification of pyridoxal kinase [57]. In this work, we successfully employed commercially available human pyridoxal kinase to achieve dAPLP by avoiding those toilsome and time-consuming procedures of enzyme purification. Thus, our method provides an easier way than the previous report to synthesize dAPLP. dAPLP is characterized by nuclear magnetic resonance (NMR) and mass spectroscopies (see Supplemental Information, Figures S1 and S2).

### 2.2. Reaction of dAPLP with 5,6-LAM

**Figure 2:**
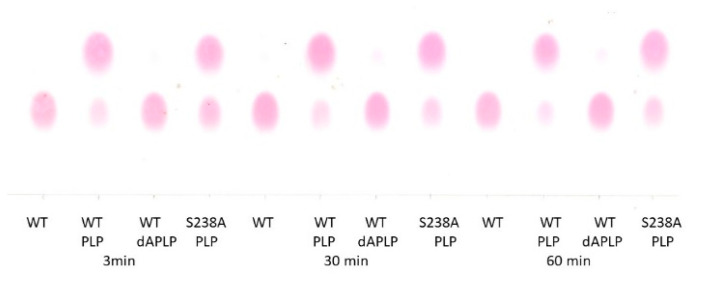
TLC chromatogram of reaction of D-lysine with WT, PLP/WT, dAPLP/WT and PLP/S238A in the presence of dAdoCbl. The time intervals were chosen at 3 min (**left**), 30 min (**middle**), and 60 min (**right**), respectively. Bottom spots are of D-lysine, whereas top spots represent the product 2,5-diaminohexanoic acid. Thus, WT and dAPLP/WT did not show any turnover, while PLP/S238A showed significant but less turnover than PLP/WT.

**Figure 3:**
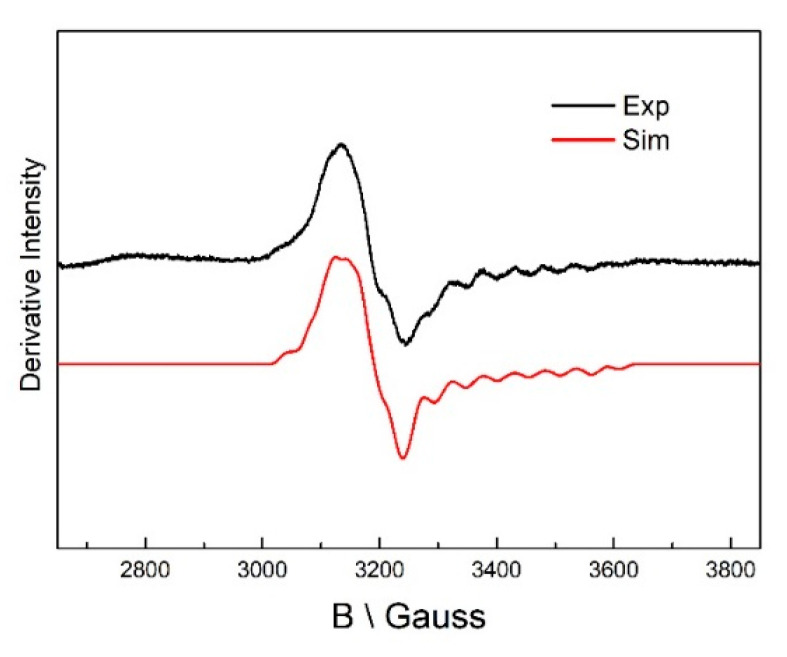
EPR spectrum in the reaction with 4-thialysine with dAPLP/5,6-LAM. Experimental parameters: microwave frequency, 9.487 GHz; power, 2 mW; modulation, 8 G at 100 kHz; T = 80 K. Simulation: *g*_Co_ = [2.278 2.227 2.000], *g*_radical_ = [2.014 2.01 2.004], Euler’s angle = [22° 28° −5°]; *A*_Co_ = [20 3 110] G; *A*_N_ = 19 G; J = 8178 G; D = −168 G; E = 0; Euler angle = [31° 48° 5°]; line width = 18 G.

5,6-LAM reconstituted with dAPLP could not turn over the substrate, D-lysine, to the product, as evident from the thin layer chromatography result (TLC) (Figure 2). dAPLP/AAT and dAPLP/AR showed a >10^9^-fold and >700-fold decrease in activity compared to that of PLP/AAT and PLP/AR, respectively. In contrast, dAPLP/OASS showed an ~250-fold decrease in activity in comparison to PLP/OASS [5]. Spectroscopic investigations were performed to find out in which step the reaction is stopped in the dAPLP/wild-type (WT)-5,6-LAM. As expected, no dAPLP-lysyl radicals could be detected with D-lysine in the EPR experiment. A D-Lysine-derived substrate-related radical eludes detection as it is highly unstable. Interestingly, a substrate-related radical was observed in the reaction with 4-thia-L-lysine (Figure 3) for 4 min. The spectrum is similar, but not exactly identical, to that observed with PLP/5,6-LAM at a reaction time of 8 s with 4-thia-L-lysine. This radical was characterized as the substrate-related radical S^•^ [32]. The spectrum could be simulated using similar parameters [29]. Although g and A values are identical in both spectra, D and J_iso_ values are slightly different—D values of −168 and −180 G, J_iso_ values of 8178 and 8000 G for dAPLP and PLP [28], respectively. Similarly, the two sets of Euler’s angles are also slightly different [28]. Hence this spectrum obtained with dAPLP can be attributed to the corresponding S^•^, but the relative orientation of radicals (S^•^ and Co(II)) is slightly different than that observed in the case of PLP/5,6-LAM. Taken together, this suggests that the initial steps of the catalytic cycle, including the transamination reaction, the protein motion that brings AdoCbl into the active site, and the Co-C bond cleavage, can be performed with dAPLP.

### 2.3. Kinetic Data of S238A Variant

As evident from the TLC results (Figure 2), the S238A variant retains significant activity in comparison to the WT. From the kinetic experiments, only a 3.2-fold decrease in catalytic turnover (*k*_cat_, Table 1) is observed for the S238A variant as compared with the wild-type 5,6-LAM, while a 4.7-fold decrease in catalytic efficiency (*k*_cat_/*K*_m_) is observed for D-lysine. On the contrary, the reduction in activity observed for the S162A variant of 4,5-OAM [58], the S272A variant of OASS [38], and the S377A variant of β-TRPS [40] were 22-fold, 80-fold, and ~300-fold, respectively. It is surprising that the serine-to-alanine mutation is much less deleterious in 5,6-LAM than 4,5-OAM. The kinetic isotope effects (KIEs) upon the reaction with D-lysine-4,4,5,5-d_4_ were measured. A 2.7-fold increase in ^D^*k*_cat_ and a 1.9-fold increase in ^D^*k*_cat_/*K*_m_ in S238A, as compared to the wild-type 5,6-LAM (Table 1), suggests that hydrogen atom transfer becomes more rate-determining in the case of S238A than the wild-type. On the contrary, mutation of the conserved serine of 4,5-OAM resulted in a reduction in KIEs on ^D^*k*_cat_ and ^D^*k*_cat_/*K*_m_ compared to the wild-type 4,5-OAM [58]. This suggests that a step that does not involve hydrogen transfer has become more rate-determining in the case of this variant than the wild-type 4,5-OAM. In the case of 4,5-OAM, a more pronounced reduction of ^D^*k*_cat_/*K*_m_ than ^D^*k*_cat_ demonstrates that substrate binding or release is more rate-determining, as *k*_cat_/*K*_m_ relates to the substrate binding and release. Although 4,5-OAM and 5,6-LAM catalyze similar reactions, a contrasting KIE of the 4,5-OAM variant in comparison to the 5,6-LAM variant is of particular interest; it may be explained by the differing nature of the active site, with 5,6-LAM being less compact in comparison to 4,5-OAM and, thus, behaving differently in some aspects (see later).

### 2.4. Stopped-Flow Analysis

**Figure 4:**
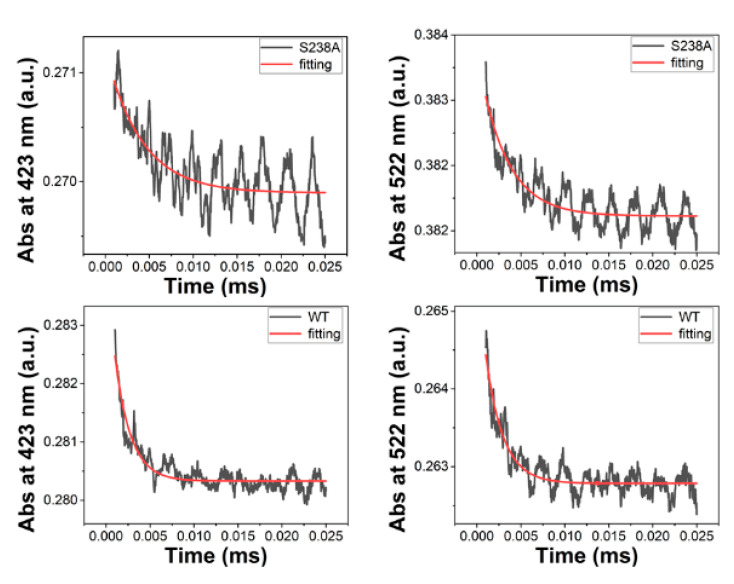
Stopped-flow absorbance changes (black) following transaldimination and homolysis in the S238A variant (**top panel**) and WT 5,6-LAM (**bottom panel**). Transaldimination (**left panel**) was monitored at 423 nm. Homolysis (**right panel**) was monitored at 522 nm. The rate constant was extracted with a fitted single-exponential decay function (red).

Stopped-flow analysis reveals the effect of the serine-to-alanine mutation on transaldimination, Co-C bond homolysis, and suicide inactivation in the reaction of 5,6-LAM (Figure 4 and Figure S3). As shown in Figure 4, the rate constants for transaldimination are 237 and 495 s^−1^ for the S238A and WT, respectively, whereas those for homolysis are 262 and 481 s^−1^ for the S238A and WT, respectively. S238A elicits a ~2-fold decrease in the rate of both transaldimination, and Co-C bond homolysis is observed with respect to that of the wild-type 5,6-LAM. That the rate of transaldimination is about the same as that of homolysis means the cleavage of the Co-C bond is gated by the substrate binding. These rate reductions corroborate well with 3.2-fold decrease in catalytic turnover in the case of Ser238A. To find the rate of suicide inactivation, the rate of hydroxocobalamin formation was followed at 356 nm. Like transaldimination and Co-C bond homolysis, a 5.3-fold decrease in the rate of suicide inactivation was observed as compared to WT (Figure S3), which further corroborates the kinetic parameters.

### 2.5. EPR investigation with S238A Variant

**Figure 5:**
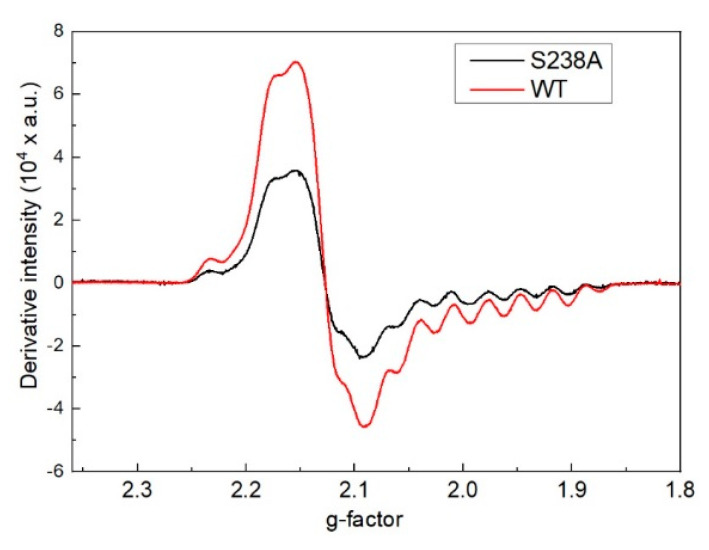
EPR spectra of S238A variant reaction with 4-thial-L-lysine at reaction time 8 s. EPR spectra of WT (red) and S238A (black) showing decreased signal intensity. The features of both spectra are identical. Experimental parameters: microwave frequency, 9.467 GHz; power, 2 mW; modulation, 8 G at 100 kHz; T = 80 K.

As expected, no PLP-lysyl radical was detected with D-lysine in the EPR experiment. Interestingly, a substrate-related radical is observed in the reaction of the S238A variant with 4-thia-L-lysine at a reaction time of 8 s (Figure 5). The features of the spectra are identical to that observed with wild-type 5,6-LAM, with the exception of the intensity being slightly less in the case of S238A variant. This demonstrates that the active-site geometry is not altered in spite of the mutation of the serine residue to alanine. That the intensity of the EPR signal for S238A is approximately half of that for wild-type 5,6-LAM corroborates well with the results obtained from the stopped-flow experiments.

### 2.6. DFT Calculations

**Scheme 3:**
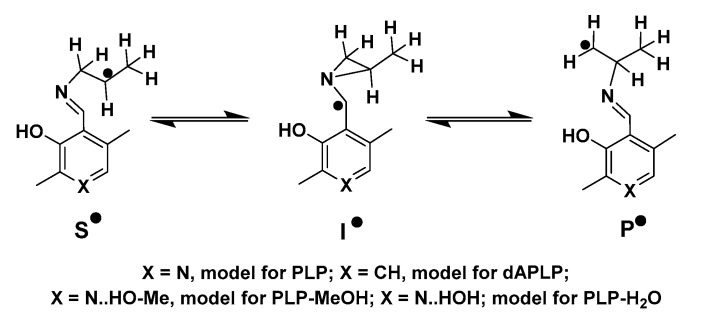
Model of radical intermediates in the reaction of 5,6-LAM with lysine.

**Figure 6:**
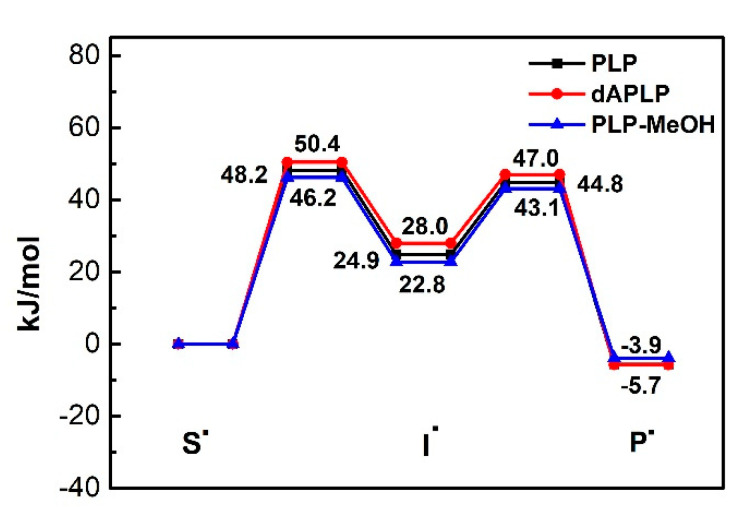
Relative energies of models of important radical intermediates in the reaction of 5,6-LAM.

DFT calculations were performed on the models of PLP, dAPLP, PLP-MeOH and PLP-H_2_O in the gas phase using established strategies (Scheme 3). The energies were calculated at the level of RMP2/G3MP2Large and were corrected for zero-point energy. Figure 6 and Table 2 display the relative energies of the important radical intermediates shown in Scheme 3.

The computations suggest that the replacement of nitrogen with carbon results in slight destabilization of the intermediate radical by ~3 kJ mol^−1^. The extent of destabilization energy due to the replacement of nitrogen with carbon is not significant. This suggests a very minor contribution. It is worth noting that previous reports suggest that with the protonated PLP, this difference becomes vast [53]. In 5,6-LAM, protonated PLP is not feasible, as Ser238 is a weak acid. Instead, Ser238 forms a hydrogen bond with the nitrogen atom of PLP. This can be thought of as a partial proton transfer to the nitrogen of PLP. The role of partial proton transfer as a facilitator for catalysis by stabilizing the intermediate radical has been demonstrated in coenzyme B_12_-dependent enzyme methylmalonyl-CoA mutase [59,60]. By analogy, a partial proton transfer would stabilize the cyclic intermediate radical at an optimum level so that the reaction proceeds. A fully protonated nitrogen may over-stabilize the intermediate and hamper the reaction progress. Therefore, a partial proton transfer may be crucial for radical aminomutases. The relative energies calculated on PLP-MeOH models the PLP/S238A interaction and the effect of this partial proton transfer. It stabilizes the cyclic intermediate radical by ~2.1 kJ mol^−1^ with respect to the PLP. This means that the replacement of PLP by dAPLP would destabilize the cyclic intermediate radical by a total of ~5 kJ mol^−1^. The calculations also suggest a higher activation barrier for a cyclic intermediate formation of merely ~4 kJ mol^−1^ for dAPLP, with respect to hydrogen-bonded PLP. Therefore, these electronic factors may contribute partially, but alone, cannot explain the total loss of activity of dAPLP/5,6-LAM. We note that the relative energies of PLP-H_2_O (not shown in Figure 6 for clarity) and PLP-MeOH are almost identical (Table 2).

### 2.7. Role of the Nitrogen Atom of PLP

5,6-LAM reconstituted with dAPLP does not show any detectable activity. This demonstrates that the nitrogen atom of PLP is essential for the catalysis of radical aminomutases. The replacement of a nitrogen atom with a carbon atom results in a simultaneous loss of hydrogen-bonding interaction with the amino acid residue of the enzyme. The contribution of hydrogen bonding consists of electronic contribution via partial proton transfer and structural contribution by keeping the PLP, PLP-bound substrate, PLP- bound reaction intermediates, and PLP-bound product in the appropriate orientation for substrate binding, the progress of reaction, and finally, the release of the product, respectively. Less favorable binding of the substrate might partially contribute to the loss of activity in dAPLP/5,6-LAM. However, the UV–Vis and EPR data with 4-thialysine suggest that dAPLP/5,6-LAM has the ability to proceed at least until the formation of a substrate-based radical. The gas-phase computations suggest that the electronic contribution of hydrogen bonding to (the partial protonation of) nitrogen for catalysis in radical PLPoenzymes is not very significant. Nevertheless, quantum mechanics and molecular mechanics (QM/MM) calculations on 4,5-OAM revealed that the orientation of the ring of a cyclic intermediate radical in an enzymatic environment was different to that of a gas phase [51]. The interactions of the PLP-bound intermediate radical with active-site residues imposed a constrained active-site geometry to access this rather higher-energy but productive orientation of the ring, so that succeeding steps to complete the catalytic cycle were achieved. The authors estimated an increase in strain energy by ~36 kJ mol^−1^ going from the substrate radical to the cyclic intermediate radical, and argued that the electrostatic interactions with Arg297 and Glu81 were major contributor to this. This resulted in a more obvious increase in the activation barrier for the ring closure inside the enzymatic environment than in the gas phase. A similar situation is expected in the case of 5,6-LAM, with the electrostatic interactions between Asp298α and Lys370α being the major contributor. Active-site residues that interact with PLP are highly conserved between 5,6-LAM and 4,5-OAM, with the exception of one additional hydrogen-bonding interaction with His225 in the case of 4,5-OAM. However, the computations revealed that the impact of the hydrogen bonding of PLP with His225 and Ser162 is negligible on the barrier heights of the reaction profile of 4,5-OAM in the gas phase [51]. Interestingly, mutations of His225 to glutamine and alanine result in a 3- and 10-fold reduction in catalytic turnover, respectively [61]. These mutants revealed an enhancement of the stabilization of the radical intermediate when reacted with the inhibitor 2,4-diaminobutyric acid. In other words, in these mutants of 4,5-OAM, the formation of a productive azacyclopropylcarbinyl radical becomes less favorable. Taken together, His225 in 4,5-OAM plays a key role by forming a hydrogen-bonding interaction with the phenolic hydroxyl group of PLP to create the optimum architecture of the active site, such that the corresponding azacyclopropylcarbinyl radical can be oriented in a productive fashion. The hydrogen-bonding interaction between the nitrogen atom of PLP and the serine residue may play a vital role in keeping the active site compact, and may contribute significantly to the requisite strain energy for the formation of an optimally oriented cyclic intermediate for further progress of the reaction. The absence of a similar hydrogen bonding interaction of PLP with histidine in 5,6-LAM renders the hydrogen-bonding interaction between the serine and nitrogen of PLP even more critical for catalysis. This crucial hydrogen bonding interaction is absent in dAPLP/5,6-LAM, and results in a relaxed active site, which is not conducive to the formation of an optimally oriented cyclic intermediate. Thus, the nitrogen atom of PLP is indispensable for radical catalysis in radical aminomutases.

Although the loss of hydrogen bonding in the case of dAPLP/5,6-LAM results in a loss of activity, very little impact on the activity of PLP/S238A seems apparently anomalous. The established notion in PLP-dependent enzymology is that the mutation of the serine residue to alanine results in the loss of hydrogen bonding. Accordingly, it is expected that S238A should result in a huge loss of activity, such as in the case of dAPLP/WT, as it is discussed above that the hydrogen bonding between PLP and serine is crucial for catalysis. Interestingly, the EPR results demonstrate that the active-site geometry is almost identical in the wild-type and Ser238Ala. The most obvious effect of the absence of hydrogen bonding should be the change in the orientation of the active-site geometry. That is what happened in the case of dAPLP, as is evident from similar, but slightly different, EPR spectra. On the basis of these two results, we hypothesize that in Ser238Ala, somehow, PLP nitrogen is still hydrogen-bonded to either an amino acid residue or a water molecule. Although a cysteine residue, which is capable of forming a hydrogen bond through a thiol moiety, is in the vicinity of PLP nitrogen in the active site of 5,6-LAM, its side chain is oriented away from PLP in the wild-type crystal structure [24]. So, we propose that a water molecule forms a hydrogen bond with the pyridine nitrogen of PLP in the S238A variant of 5,6-LAM. In fact, the mutation of serine to alanine can be conceived as the generation of a cavity in the active site due to an oxygen atom vacancy. A water molecule should exactly fit into this oxygen cavity, as the active site of 5,6-LAM is known to be flexible. It was shown that even an extra oxygen atom can be accommodated in the active site of the wild-type, as demonstrated by the turnover of the substrate in PLP-NO/5,6-LAM [46]. DFT calculations suggest, as shown in Table 2, that a water molecule hydrogen-bonded to PLP should have an almost identical energy profile to a serine molecule hydrogen-bonded to PLP for a relevant radical intermediate along the reaction path. Moreover, the crucial role played by active-site water molecules is not unprecedented in PLPoenzymes.

Strong support for our hypothesis comes from the fact that a water molecule is, indeed, hydrogen bonded to the nitrogen atom of PLP in 2,3-LAM, which is a dAdo- and PLP-codependent enzyme like 5,6-LAM and has a similar azacyclopropylcarbinyl radical as an important [36] intermediate in the reaction mechanism. Moreover, a network of hydrogen bonds involving a specific water molecule plays key role in stabilizing the negative charge of quinonoid intermediate in the case of PLP-dependent histidine decarboxylase (HDC) [62]. This specific water molecule forms a hydrogen bond with an aspartate residue that directly interacts with the pyridine nitrogen of PLP. Active-site-bound water molecules were shown to facilitate the catalytic reaction in PLP-dependent serine hydroxymethyltransferase (SHMT) [63]. The pyridine nitrogen of nicotine forms a hydrogen bond with a water molecule in the water-soluble acetylcholine-binding protein (AChBP) [64] and α3β4 nicotine acetylcholine receptor (nAChR) [65]. A similar functionally important hydrogen bond between pyridine nitrogen and backbone NH, mediated through a water molecule, was proposed in the case of α4β2 nAChR by combining a mutagenesis and analog binding investigation, in which the pyridyl ring of nicotine was replaced by a phenyl ring [66]. On the basis of these, we propose that a water molecule is hydrogen-bonded to the pyridine nitrogen of PLP in S238A.

This hypothesis would also explain the apparent anomaly of KIEs between 5,6-LAM and 4,5-OAM. The results of the DFT computations demonstrate that the relative energy level of I^•^ remains unaltered if a water molecule substitutes a serine residue as the hydrogen-bonding partner (Table 2). This suggests that the transformation of S^•^ to I^•^ has no impact on the deuterium KIE. Nevertheless, the slight conformational change in the substrate-PLP-H_2_O relative to the substrate-PLP-S238 would make the H-atom abstraction step a little bit difficult and, thus, would contribute to the increase in KIEs. On the other hand, in the compact active site of 4,5-OAM, a water molecule is unlikely to fit in the S162A variant of 4,5-OAM. This means there would be absence of H-bonding to the nitrogen atom of PLP. This would raise the energy of I^•^, making the formation of it more rate-limiting, and would result in the decrease in the deuterium KIEs in the S162A of 4,5-OAM.

**Figure 7:**
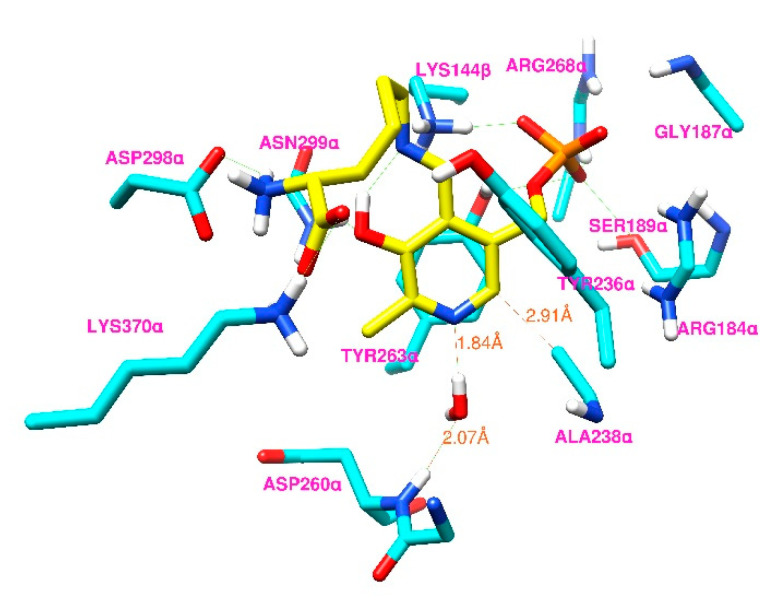
Stereo view of the active-site interactions of D-lysyl-PLP-H_2_O docked into the open state of the S238A variant of 5,6-LAM. Selected residues of 5,6-LAM (cyan) are shown in stick representation. D-lysyl-PLP (yellow) and water are also shown in stick representation.

To assess the feasibility of our hypothesis, we performed docking of D-lysyl-PLP that is hydrogen-bonded to a water molecule (D-lysyl-PLP-H_2_O) into the open state of an S238A model of 5,6-LAM (Protein Data Bank (PDB) ID code 1XRS). The docked conformer with the lowest energy among the 100 docked conformers was chosen as the best binding mode. The active-site arrangement of the best binding mode of D-lysyl-PLPH_2_O is shown in Figure 7.

The binding mode of docked D-lysyl-PLP-H2O into the S238A of 5,6-LAM is similar to that of docked D-lysyl-PLP into WT 5,6-LAM, which has been reported before [34]. The carbon atom of Ala238 is still within ~3 Å from the pyridine ring. A new hydrogen-bonding interaction is evident, involving the water molecule and backbone NH of Asp260 to anchor the water molecule in a fixed place, such that it can also form hydrogen bonding with the pyridine nitrogen of PLP. The distance between the oxygen atom of the water molecule and the amide hydrogen of Asp260 is 2.07 Å. Similar interactions involving backbone amide hydrogen atoms with a water molecule hydrogen-bonded to PLP is present in the crystal structure of 2,3-LAM [36]. Thus, a water molecule can act as an anchor to keep D-lysyl-PLP complex in the suitable orientation in the S238A variant for catalysis to proceed. This specific water molecule mediates the interaction between PLP and the enzyme backbone to keep the active site, such that the optimal orientation of the cyclic intermediate is still accessible for the progress of the reaction. Moreover, this specific water molecule also rescues the partial proton transfer, which may partially contribute to stabilizing the cyclic intermediate radical, as discussed above. We note that this is the first instance, to the best of our knowledge, of a water-mediated rescue of enzymatic activity in a variant in which a conserved serine residue is mutated to alanine.

The active site of 4,5-OAM is more compact than 5,6-LAM due to the presence of the additional interaction between PLP and His225, introducing additional restraint in the active-site architecture. The superimposition of the substrate-free crystal structure of both reveals that the Asp298 of 5,6-LAM (PDB ID code 1XRS) overlays with the His225 of 4,5-OAM (PDB ID code 3KP1), but does not form any polar interactions with PLP (Figure S4). The absence of the bulky imidazole ring of histidine in 5,6-LAM obviously renders the active-site architecture less compact. Accepting a water molecule, if any, would evidently be less favorable for the Ser162A variant of 4,5-OAM than for the Ser238A variant of 5,6-LAM. Thus, a water-mediated rescue of activity would obviously be less efficient in the case of 4,5-OAM, resulting in a higher reduction in activity than that of 5,6-LAM. Thus, our results suggest that an optimal hydrogen-bonding interaction between the nitrogen atom of PLP and the enzyme backbone is crucial for catalysis; this is mediated through either a conserved serine residue in the case of 5,6-LAM and 4,5-OAM, or a water molecule in the case of 2,3-LAM and the Ser238Ala variant of 5,6-LAM.

## 3. Materials and Methods

Human pyridoxal kinase was purchased from ATGen, NKMAX, Gyeonggi-do, Korea (Catalog No. ATGP0777). D-lysine-4,4,5,5-*d*_4_ was purchased from CDN Isotopes, Pointe-Claire, QC, Canada. Other chemicals were purchased from Sigma-Aldrich, St. Louis, MO, US, Acros, Geel, Belgium, TCI, Tokyo, Japan, and Alfa-Aesar, Haverhill, MA, US. NMR spectra were obtained using 400 MHz Bruker spectrometer. Mass spectra were measured using Bruker. UV–visible spectra were measured using Shimadzu spectrometer.

### 3.1. Synthesis of dAPLP

1-deazapyridoxine was synthesized via an initial five steps according to the literature procedure and characterized by NMR [47,67]. All the operations for the following two steps were performed either under dark or red light.

1-deazapyridoxine (202 mg, 1.20 mmol), sodium acetate (492 mg, 6.00 mmol), and powdered molecular sieve (500 mg) were taken, under an argon atmosphere, in a 3-necked 50 mL round-bottomed flask, and covered with aluminum foil. Then, ethyl acetate (14 mL), previously degassed by purging argon gas for 45 min after keeping over a molecular sieve for 3 days, was added to this mixture. A solution of pyridinium chlorochromate (PCC, 162 mg, 0.75 mmol) was prepared using 14 mL degassed ethyl acetate, and was added to the previous mixture through the septum using a syringe. The reaction mixture was stirred under argon, in the dark, at room temperature, for one hour. Another batch of PCC (162 mg, 0.75 mmol) was added to the reaction mixture and was stirred for another hour. Then, the reaction mixture was filtered and washed with 20 mL ethyl acetate: ether (1:1). The solvent of the filtrate was removed. The residue obtained, thus, was chromatographed on silica gel using ethyl acetate/hexane (1:3) followed by ethyl acetate/hexane (1:1) to separate and recover 1-deazapyridoxine. 2,4-dinitrophenylhydrazine (DNP) positive fractions were combined together and the solvent was removed. Then it was dissolved in KOH, and this solution was used, as such, for the next step. The concentration was estimated [47] by measuring the absorbance at 344 nm using the extinction coefficient of salicylaldehyde, 3300 M^−1^ cm^−1^ of 1-deazapyridoxal; using UV–visible spectroscopy revealed a yield of 16%.

The solution containing 1-deazapyridoxal was phosphorylated by 1 μM human pyridoxal kinase (ATGen) in 100 mM NH_4_^+^EPPS buffer at pH 8.5, 1 mM 1-deazapyridoxal, 4 mM MgATP and 10 mM KCl. The mixture was stirred slowly in a tinted-glass round-bottomed flask overnight at room temperature. Then, the reaction mixture was quenched with the addition of 5% trichloroacetic acid. After the removal of protein precipitates, the mixture was chromatographed on a C18 5μm reverse-phase column (Waters, 4.6 × 250 mm) using high-performance liquid chromatography (HPLC), which was pre-equilibrated with buffer A (10 mM ammonium carbonate), using a linear gradient elution with a flow rate of 1 mL/min, and monitored at 344 nm. The gradient was as follows: 0–5 min, 0% buffer B (80% acetonitrile/water); 5–15 min, 0–10% buffer B; 15–25 min, 10% buffer B; 25–40 min, 10–25% buffer B; 40–50 min, 25% buffer B. With this gradient, dAPLP had an elution time of 19.8 min. The eluent containing dAPLP was collected together, repeatedly lyophilized to remove ammonium carbonate, and stored at −20 °C. Yield = 56%. dAPLP was characterized by identical ^1^H NMR spectra, as reported earlier, and high-resolution mass spectroscopy (HRMS) (Calculated for [M-H]^−^: 245.0215; observed: 245.0214); both the spectra are provided in the Supplemental Information (Figures S1 and S2). It is worth noting that electrospray ionization (ESI) mass spectroscopy in negative mode produced the desired peaks for dAPLP, while positive mode, which is usually used for PLP and analogues, did not show the desired peaks.

### 3.2. Purification of 5,6-LAM and S238A Variant

The recombinant 5,6-LAM from *Clostridium sticklandii* was produced using expression in *Escherichia coli* and purified following the procedures described by Chang and Frey [68]. Twelve grams of cells was lysed using suspension and sonication after frozen cells were thawed in 30 mL of 50 mM potassium phosphate, pH 7.0; 0.5 mM ethylenediaminetetraacetic acid (EDTA); 0.5 mM phenylmethylsulfonyl fluoride (PMSF); and 2 mM β-mercaptoethanol (β-ME). To the crude extract was added 0.1% of polyethylenimine, which was centrifuged at 12,000× *g* for 45 min to precipitate the insoluble component. The supernatant was loaded onto a Q-Sepharose Fast-Flow column (2.5 × 30.5 cm) equilibrated with 20 mM potassium phosphate, pH 7.0, and 2 mM β-ME, and attached to an ÄKTA pure protein purification system, GE Healthcare Life Sciences Inc. The column was washed with equilibration buffer. The enzyme was eluted using an increasing gradient of KCl (0.0–0.6 M) in 1360 mL of buffer in 20 mM potassium phosphate, pH 7.0, and 2 mM β-ME, at a flow rate of 2 mL min^−1^. The fractions with peak activity were collected and concentrated. The concentration of KCl was diluted to 20 mM using equilibration buffer. All purification steps were performed at approximately 6 °C. The 5,6-LAM concentration was determined using the extinction coefficient ε_280_ = 60,345 M^−1^ cm^−1^ [69].

Site-directed mutagenesis was performed using the Phusion High-Fidelity DNA Polymerase (Finnzymes, Finland) and the following primers (Mission Biotech Co., Ltd., Taipei, Taiwan): S238A Forward Strand, 5′-TACTGTGCTGGTTTATGTATGCCTG-3’; S2388A Reverse Strand, 5′-TAAACCAGCACAGTAATTACAAAGTC-3’. Purification of S238A variant was performed similarly to 5,6-LAM.

### 3.3. Kinetic Parameters

The kinetic parameters of 5,6-LAM and S238A variant were measured based on HPLC analysis of phenylisothiocyanate (PITC)-derivatized D-lysine and 2,5-diaminohexanoic acid (2,5-DAH). 5,6-LAM (5 μM) and S238A (20 μM) were incubated with dAdoCbl and PLP—with a concentration 1.25 times that of the enzyme—and 5 mM β-ME in 100 mM NH_4_-EPPS buffer at pH 8.5, in a total volume of 50 μL, for 30 min, on the ice. The reaction was initiated by the addition of various concentrations of D-lysine (6, 8, 10, 15, 25, 40, 70, and 140 mM). The reaction was allowed to proceed at 37 °C for different time periods (15, 30, 45, and 60 s), quenched by adding HClO_4_, and centrifuged. The supernatant was loaded onto a C18 Sep-Pak cartridge (Waters) to remove 5′-deoxyadenosine and dAdoCbl. The substrate D-lysine and product 2,5-DAH were eluted with deionized water. D-lysine and 2,5-DAH were derivatized through a reaction with PITC, as described previously [70]. PITC-derivatized D-lysine and 2,5-DAH were applied to a C18 column (XBridge, Waters) for HPLC analysis. For deuterium KIE experiments, the enzyme concentrations of 5,6-LAM and S238A were increased to 20 μM and 125 μM, respectively, and concentrations of D-lysine-4,4,5,5-*d*_4_ were adjusted to 6, 8, 10, 15, 25, 40, and 70 mM. The experimental steps were the same as above.

### 3.4. EPR Spectroscopy

X-band EPR spectra were collected using a Bruker EMX spectrometer equipped with a Bruker TE_102_ cavity. The temperature was controlled by an Advanced Research System Helitran continuous-flow cryostat. 5,6-LAM or the S238A variant (0.65 mM) were incubated with 100 mM NH_4_ EPPS buffer at pH 8.5; dithiothreitol (2 mM); dAdoCbl (0.81 mM); and PLP (0.81 mM) for 30 min on the ice. The reactions were initiated by adding 4-thia-L-lysine (65 mM), an inhibitor, to the holoenzyme mixture [29]. The mixtures (~0.2 mL) were allowed to react in an EPR tube for 8 s (PLP) and 4 min (dAPLP) to obtain the substrate-related radical. Then, they were frozen immediately in liquid nitrogen-chilled ethanol and stored in liquid nitrogen for the EPR measurement. Simulations of the EPR spectra were performed using EasySpin [71].

### 3.5. Stopped-Flow Analysis

Anaerobic stopped-flow studies were conducted under red-light conditions in a Coy vinyl anaerobic chamber (O_2_ concentration <5 ppm). The holoenzyme and substrate solutions were mixed (1:1) in a stopped-flow spectrophotometer (SX20, Applied Photophysics). Anaerobic solutions of the cofactors, substrate and buffer were prepared by purging argon gas for 1 h. The holoenzyme solution was prepared by mixing 60 μM apoenzyme and equimolar amounts of PLP and dAdoCbl in NH_4_EPPS buffer (100 mM, pH 8.5), and followed by incubation on ice for 30 min. The substrate solution of D-lysine (12 mM) in NH_4_EPPS (100 mM, pH 8.5) was used for this purpose. Pre-steady-state analysis was performed at ambient temperature and the spectra were recorded using the photomultiplier (R928, Applied Photophysics). The results were based on the average of seven shots, with each shot containing 1000 data points in 25 ms. The absorbance traces at 522 or 423 nm were fitted with the single exponential decay function: y = A e^−k t^ + c.

### 3.6. DFT Calculation

All the computations were performed using the Gaussian 09 program package [72] following the established protocol for the reactions catalyzed by radical aminomutases [46,49,52,53,54,55]. In order to save computation time, lysine was modelled as 1-aminopropane, and the methylene phosphate of PLP and dAPLP was replaced by a methyl group, as the phosphate group is believed not to be essential for catalysis. Computations were also performed on PLP that was hydrogen bonded to a methanol (MeOH-PLP) and water (H_2_O-PLP), which modeled the hydrogen bonding between PLP and a serine residue of the enzyme and a water molecule, respectively. Geometry optimizations and calculations of zero-point vibrational energies were performed in gas phase at the level of B3LYP/6-31G(d,P), and improved energies were obtained by using RMP2/G3MP2Large procedure and corrected for zero-point energy, after scaling with a factor of 0.9806. The adequacy of these models has been validated with experimental results [52]. This level of consistency lends significant confidence to the use of the less expensive techniques for the quantitative evaluation of larger systems.

### 3.7. Substrate Docking Modeling

The docking simulations were conducted using AutoDock version 4.2.6.1 and AutoDock Tools version 1.5.6, obtained from the Scripps Research Institute (La Jolla, CA, USA) [73]. In the crystal structure of 5,6-LAM holoenzyme, PLP was bound to Lys144β. The substrate formed an imine linkage with PLP, replacing Lys144β to initiate catalysis. For docking purposes, PLP was removed from the crystal structure of 5,6-LAM (PDB ID code 1XRS) using GaussView, and only one of the α and β chains were retained. Then, it was saved as a pdb file and treated as input for a macromolecule. A model of the Ser238Ala variant was generated using the rotamer tool for UCSF Chimera [74]. Hydrogen atoms were added to the PLP-depleted structure (receptor) and Gasteiger charges were added to each atom. D-lysyl-PLP-H_2_O adduct, in which D-lysyl-PLP was hydrogen-bonded to a water molecule, was prepared as the ligand. The coordinates of the phosphate handle of PLP were taken directly from the original PDB file, while the remaining bond lengths and angles of the substrate–PLP adduct were energy-optimized at the B3LYP level with the 6-31G(d,p) basis using Gaussian. The hydrogen bonding between the aldimine nitrogen and the hydroxyl group of the pyridine was constrained, while all other possible torsions were set free to perform flexible ligand docking. The deficient charges were spread over the entire ligands after computing Gasteiger charges. The size of the grid box was set such that it encompassed the site of the original PLP cofactor, residues that interacted with PLP, and a fully extended PLP–substrate adduct. One hundred structures were generated using the Lamarkian genetic algorithm. The number of energy evaluations was set to 25,000,000. Finally, the active-site interactions were visualized using UCSF Chimera [74].

## 4. Conclusions

In summary, we synthesized dAPLP by designing a simpler strategy. The inability of 5,6-LAM reconstituted with dAPLP to turn over the substrate demonstrates that the nitrogen atom of PLP is crucial for catalysis, in which hydrogen bonding between the nitrogen atom and the Ser238 residue acts as the facilitator. In contrast, the S238A mutant retains significant activity. On the basis of insights into dAPLP, we propose that a water molecule interacts with the nitrogen atom of PLP through hydrogen bonding in the S238A mutant; consequently, it rescues the activity that would have been lost due to the absence of the hydrogen-bonding interaction as a result of the mutation of serine to alanine. This hydrogen-bonding interaction between PLP and the enzyme backbone is vital for the catalysis of radical aminomutases such as 5,6-LAM; 4,5-OAM; and 2,3-LAM. In light of our result, it would be interesting to investigate whether a water-mediated rescue of the hydrogen bond between the nitrogen of PLP and the backbone of the enzyme is unique in the Ser238Ala variant of 5,6-LAM, or rather, prevalent in PLPoenzymes. This work implies that a combined study involving dAPLP, a cofactor analog, and the relevant mutagenesis investigations is required to obtain full knowledge of the role played by the nitrogen atom of PLP.

## Figures and Tables

**Table 1:** Comparison of kinetic parameters of WT and S238A variant and deuterium kinetic isotope effects upon reaction with D-lysine-4,4,5,5-d_4_.

	*K*_m_ (mM)	*k*_cat_ (s^−1^)	*k*_cat_/*K*_m_
	D-lysine		
WT	12.45 ± 0.89	20.11 ± 1.30	1.616 ± 0.043
S238A	18.14 ± 0.70	6.23 ± 0.34	0.343 ± 0.010
	D-lysine-4,4,5,5-d_4_		
WT	11.58 ± 0.84	5.13 ± 0.27	0.444 ± 0.021
S238A	12.28 ± 0.41	0.60 ± 0.02	0.049 ± 0.002
		Deuterium kinetic isotope effects (D-lysine-4,4,5,5-*d*_4_)	
		^D^ *k*_cat_	^D^(*k*_cat_/*K*_m_)
WT		3.9 ± 0.3	3.6 ± 0.2
S238A		10.4 ± 0.6	7.0 ± 0.4

**Table 2:** Relative energies of important intermediate radicals and transition states in kJ/mol.

	S^•^	TS1	I^•^	TS2	P^•^
PLP	0	48.2	24.9	44.8	−5.7
dAPLP	0	50.4	28.0	47.0	−5.7
PLP-MeOH	0	46.2	22.8	43.1	−3.9
PLP-H_2_O	0	46.5	23.1	43.2	−4.0

## Data Availability

The data presented in this study are available on request from the corresponding author.

## Supplementary Materials

The following are available online at https://www.mdpi.com/article/10.3390/ijms23095210/s1.
