# Supplementary material for: The Nitrogen Atom of Vitamin B6 Is Essential for the Catalysis of Radical Aminomutases
Source: Int J Mol Sci. 2022 May 6;23(9):5210. doi: 10.3390/ijms23095210 (PMC9105233; doi:10.3390/ijms23095210)
Supplement: Supplementary file 1 [file ijms-23-05210-s001.zip › ijms-1677961-supplementary.pdf]

Supplemental Information

The Nitrogen Atom of Vitamin B$_6$ Is Essential for the Catalysis of Radical Aminomutases

Amarendra Nath Maity, Jun-Ru Chen, Quan-Yuan Li, Shyue-Chu Ke*
Department of Physics, National Dong Hwa University, Hualien, Taiwan 97401
*Corresponding author: e-mail address: ke@gms.ndhu.edu.tw

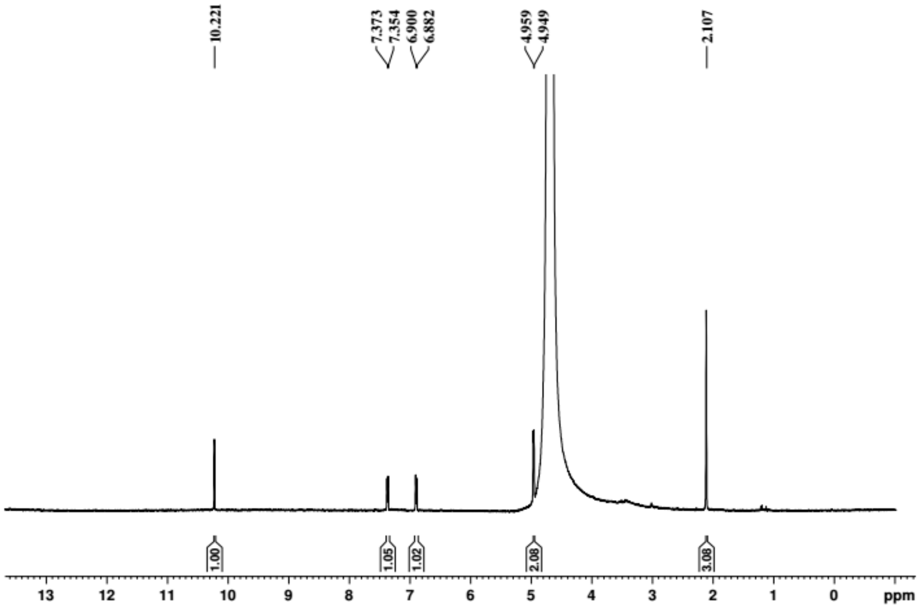

Figure S1. ^{1}H NMR spectrum of dAPLP in D_2O.

Elemental Composition Report — Page 1

Single Mass Analysis
Tolerance = 200.0 PPM / DBE: min = -20.0, max = 100.0
Element prediction: Off
Number of isotope peaks used for i-FIT = 3

Monoisotopic Mass, Even Electron Ions
234 formula(e) evaluated with 53 results within limits (up to 20 closest results for each mass)
Elements Used:
C: 1-100 H: 1-100 O: 1-10 P: 1-3 Na: 0-1
deazaPLP
190215esi01 68 (1.002) Cm (67:70)

1: TOF MS ES-
3.70e+007

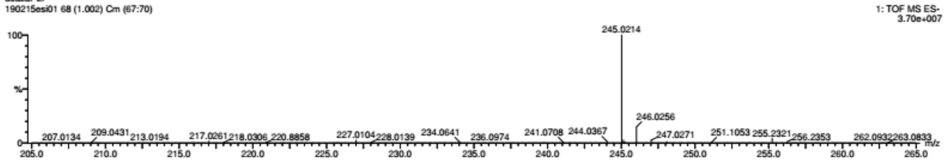

Minimum:				-20.0	
Maximum:		5.0	200.0	100.0	
Mass	Calc. Mass	mDa	PPM	DBE	Formula
245.0214	245.0215	-0.1	-0.4	5.5	C9 H10 O6 P
	245.0191	2.3	9.4	-3.5	C2 H15 O9 P2
	245.0237	-2.3	-9.4	-2.5	C4 H17 O4 P3 Na
	245.0191	2.3	9.4	2.5	C7 H11 O6 P Na
	245.0261	-4.7	-19.2	6.5	C11 H12 O P2 Na
	245.0261	-4.7	-19.2	0.5	C6 H16 O4 P3
	245.0156	5.8	23.7	14.5	C16 H6 O P
	245.0285	-7.1	-29.0	9.5	C13 H11 O P2
	245.0133	8.1	33.1	5.5	C9 H11 O4 P2
	245.0132	8.2	33.5	11.5	C14 H7 O P Na
	245.0109	10.5	42.9	2.5	C7 H12 O4 P2 Na
	245.0109	10.5	42.9	-3.5	C2 H16 O7 P3
	245.0320	-10.6	-43.3	-2.5	C4 H16 O6 P2 Na
	245.0344	-13.0	-53.1	6.5	C11 H11 O3 P Na
	245.0344	-13.0	-53.1	0.5	C6 H15 O6 P2
	245.0062	15.2	62.0	1.5	C5 H10 O9 P
	245.0368	-15.4	-62.9	9.5	C13 H10 O3 P
	245.0050	16.4	66.9	5.5	C9 H12 O2 P3
	245.0038	17.6	71.8	-1.5	C3 H11 O9 P Na
	245.0390	-17.6	-71.8	1.5	C8 H17 O P3 Na

Figure S2. HRESI Mass spectrum of dAPLP in negative ion mode.

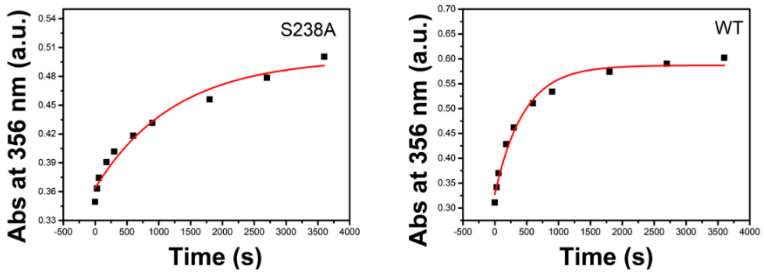

Figure S3. Comparison of rate of suicide inactivation between the S328A variant (left) and WT (right). The rate of hydroxycobalamin formation was followed at 356 nm. The suicide inactivation rates were 3.72×10^{-4} and 1.97×10^{-3} s^{-1} for S238A and WT, respectively.

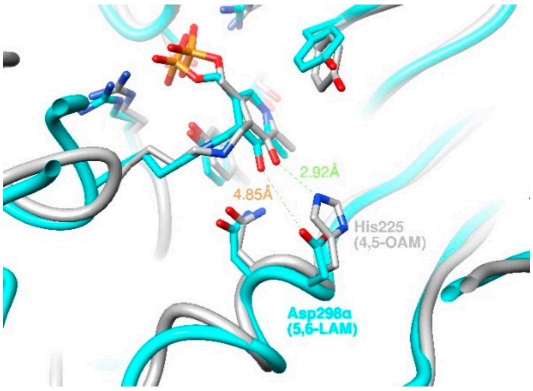

Figure S4. Superimpose of active sites of substrate free crystal structure of 5,6-LAM (cyan) and 4,5-OAM (gray). His225 of 4,5-OAM overlays with Asp298α of 5,6-LAM. His225 makes hydrogen bonding interaction with phenolic hydroxyl group of PLP whereas Asp298α is 4.85 A away from phenolic hydroxyl group of PLP indicating absence of any interaction between PLP and Asp298α.

Gaussian Archive Entries for the B3LYP/6-31G(d,P) Optimized Geometries

dAPLP

S•

1|1|UNPC-DESKTOP-TBQL3NN|SP|ROMP2-FC|GTMP2large|C12H16N1O1(2)|LABPC|12

-Nov-2020|0||# romp2 maxdisk=7840MW geom=connectivity gtmp2large scf=tight||Substrate

(1-aminopropane) radical aldimine with deaza-Pyridoxal model gtmp2 energy||0,2|N,0,-

1.72867794,0.18560711,-0.46479394|C,0,-0.75762252,1.01088273,-0.27282032|H,0,-

0.95496797,2.0895123,-0.23751674|C,0,0.6214819,0.577721,-0.0994531|C,0,0.92614231,-

0.81178441,0.12546005|C,0,2.25148205,1.27022163,0.04213924|C,0,2.96491575,1.0546402
9,0.25648006|C,0,1.66671307,1.52172431,0.09439408|H,0,3.77389137,1.76368079,0.40557
409|O,0,-0.0274474,-1.73743806,-0.30915084|H,0,-0.89850185,1.24687764,0.41260201|
C,0,-3.09470466,0.71232012,-0.59458272|C,0,-3.96067104,0.24391972,0.53537493|H,0,-
3.49810752,0.34772602,-1.54884963|H,0,-3.0783048,1.81362639,-0.63679705|H,0,-
3.86734755,0.76957773,1.4833267|C,0,-4.61036555,-1.09702047,0.52125252|H,0,-
5.4168711,-1.16614057,1.2584419|H,0,-5.02506969,-1.33366834,-0.4666265|H,0,-
3.89257975,-1.9010596,0.75752486|C,0,3.24535171,0.31890375,0.23010278|
H,0,4.27201618,-0.65301965,0.35999696|C,0,2.53221718,2.74917836,0.00963821|
H,0,1.96731351,-3.27699942,0.78576132|H,0,2.22752701,-3.18964634,-0.94608875|H,0
,3.59665046,-2.94837219,0.15953377|C,0,1.39965812,3.01073376,0.12807961|
H,0,0.95453629,3.37283914,-0.80590268|H,0,0.71978523,3.29131727,0.94102453|
H,0,2.33276497,3.558759,0.27909202||Version=IA32W-G09RevD.01|State=2-A|HF=-
593.1802553|MP2=-595.5458377|RMSD=4.902e-009|PG=C01 [X(C12H16N1O1)]||@

TS1: S•→ I•

1|1|UNPC-DESKTOP-TBQL3NN|SP|ROMP2-FC|GTMP2large|C12H16N1O1(2)|LABPC|01
-Dec-2020|0||# romp2 maxdisk=7840MW geom=connectivity gtmp2large scf=tight||
Transition State gtmp2 energy calculation for the reaction from Substrate (1-aminopropane)
radical to Intermediate radical aldimine with deAzaPyridoxal model||0,2|N,0,-1.90443531,
0.22960755,-0.61807451|C,0,-0.87562587,1.0823896,0.38947797|H,0,1.07947332,
2.15285327,-0.35389212|C,0,0.4609103,0.61811435,0.15991032|C,0,0.78019949,-
0.77520044,-0.22075326|C,0,2.08827298,-1.24141856,0.00193592|C,0,2.79799234,1.0661
1623,0.33511422|C,0,1.51291449,1.54522427,0.12098901|H,0,3.59773889,1.76901271,0.55
139531|O,0,-0.15914195,-1.70641646,-0.50069244|C,0,-3.22119349,0.10076061,
0.65210562|C,0,-3.25105293,0.75626018,-0.67129233|H,0,-3.33677163,1.84756256,-
0.68855523|H,0,-2.93039797,0.71439327,1.49824201|H,0,-3.87140889,0.29022018,-
1.43931774|C,0,-3.69264003,-1.29449341,0.89263277|H,0,-1.01444069,-1.22889812,-

0.65800231|H,0,-4.69282958,-1.29427272,1.35105252|H,0,-3.0247302,-1.83865789,
1.56858536|H,0,-3.7608004,-1.85443497,-0.04465955|C,0,2.36949434,-2.71977706,-
0.0728677|H,0,1.76155695,-3.27810697,0.64758043|H,0,2.12163012,-3.12183408,-1.061494
75|H,0,3.42362211,-2.92701628,0.13043814|C,0,1.24592943,3.03085801,0.19045343|
H,0,0.85859791,3.42526107,-0.75712049|H,0,0.51203845,3.28280541,0.9660077|
H,0,2.16613348,3.57384774,0.42007539|C,0,3.08089421,-0.30526819,0.27924828|
H,0,4.0971459,-0.65005001,0.45266054||Version=IA32W-G09RevD.01|State=2-A|HF=-
593.123119|MP2=-595.5261355|RMSD=3.435e-009|PG=C01 [X(C12H16N1O1)]||@

I•

1|1|UNPC-DESKTOP-TBQL3NN|SP|ROMP2-FC|GTMP2large|C12H16N1O1(2)|LABPC|12
-Nov-2020|0||# romp2 maxdisk=7840MW geom=connectivity gtmp2large scf=t
ight||Intermediate (1-aminopropane) radical aldimine with deaza-Pyrido
xal model gtmp2 energy||0,2|N,0,1.95688415,-0.04691219,0.33330147|C,0,
0.93209136,0.9289386,0.18480405|H,0,1.23910071,1.97287791,0.16322251|C
,0,-0.42507627,0.57066418,0.06574351|C,0,-0.86102723,-0.80262079,0.109
49673|C,0,-2.21599062,-1.14982467,-0.00689688|C,0,-2.75939209,1.218831
67,-0.21354362|C,0,-1.43019588,1.58922569,-0.10150259|H,0,-3.51477689,
1.98970865,-0.33976385|O,0,0.01736149,-1.81850548,0.2677061|C,0,3.2524
4991,0.19438784,-0.34882661|C,0,3.14931236,0.32519207,1.12631392|H,0,3
.1555612,1.32295107,1.56038037|H,0,3.29992888,1.13420508,-0.89981046|H
,0,3.53406319,-0.46075267,1.77191756|C,0,3.90814229,-0.98538424,-1.022
56584|H,0,0.92338307,-1.41872783,0.33693875|H,0,4.97415833,-0.79102032
,-1.18408122|H,0,3.44781298,-1.18493007,-1.99550159|H,0,3.81266502,-1.
88560766,-0.40892347|C,0,-2.6120642,-2.60291073,0.04708349|H,0,-2.1240
5736,-3.17823036,-0.74738659|H,0,-2.30647861,-3.06363355,0.99296362|H,
0,-3.69439259,-2.71636253,-0.0588785|C,0,-1.04508973,3.04723352,-0.155
27697|H,0,-0.5455056,3.37507833,0.76528326|H,0,-0.35809481,3.26082287,

-0.98399468|H,0,-1.93044712,3.67350615,-0.29072039|C,0,-3.15096573,-0.
12853432,-0.16775429|H,0,-4.20316658,-0.38464877,-0.25885356||Version=
IA32W-G09RevD.01|State=2-A|HF=-593.1504092|MP2=-595.5365304|RMSD=4.171
e-009|PG=C01 [X(C12H16N1O1)]||@

TS2: I$^{\bullet}$→ P$^{\bullet}$

1|1|UNPC-DESKTOP-TBQL3NN|SP|ROMP2-FC|GTMP2large|C12H16N1O1(2)|LABPC|15
-Dec-2020|0||# romp2 maxdisk=7840MW geom=connectivity gtmp2large scf=t
ight||Transition State gtmp2 energy calculation for the reaction from
Intermediate radical to Product (2-aminopropane) radical aldimine with
deAzaPyridoxal model||0,2|N,0,1.9064287,-0.13115248,-0.07679024|C,0,0
.96286002,0.84098879,-0.10766726|H,0,1.28537181,1.88218177,-0.14237957
|C,0,-0.43865894,0.54710315,-0.06203408|C,0,-0.91103825,-0.8034791,-0.
04762985|C,0,-2.28552924,-1.10218533,-0.01049003|C,0,-2.75197556,1.290
4007,-0.01675056|C,0,-1.40098056,1.60549246,-0.05127163|H,0,-3.4847318
2,2.0924235,-0.00553462|O,0,-0.06162283,-1.85292747,-0.07647067|C,0,3.
30510047,0.23895522,0.00711349|C,0,3.00721874,-0.2048819,1.38450843|H,
0,2.69257324,0.50987788,2.133539|H,0,3.48198858,1.31648657,-0.09529497
|H,0,3.14588756,-1.23880547,1.67376163|C,0,4.24237064,-0.5878337,-0.85
082906|H,0,0.86091779,-1.49110716,-0.11800227|H,0,5.28235164,-0.404605
51,-0.56211754|H,0,4.12476474,-0.33244304,-1.90828946|H,0,4.03153991,-
1.65464142,-0.73170868|C,0,-2.72868304,-2.54202767,0.00452452|H,0,-2.3
6689918,-3.0780571,-0.87978347|H,0,-2.32483143,-3.07373124,0.87318573|
H,0,-3.8193268,-2.61455978,0.03102912|C,0,-0.96927256,3.05346776,-0.07
406203|H,0,-0.3407276,3.31075776,0.78735221|H,0,-0.3936826,3.29828111,
-0.97525987|H,0,-1.84116752,3.71194409,-0.05290885|C,0,-3.18703146,-0.
04201215,0.00583758|H,0,-4.25232805,-0.25644412,0.03421155||Version=IA
32W-G09RevD.01|State=2-A|HF=-593.123735|MP2=-595.5272649|RMSD=5.630e-0

09|PG=C01 [X(C12H16N1O1)]||@

P•

1|1|UNPC-DESKTOP-TBQL3NN|SP|ROMP2-FC|GTMP2large|C12H16N1O1(2)|LABPC|11
-Dec-2020|0||# romp2 maxdisk=7840MW geom=connectivity gtmp2large scf=t
ight||Product (2-aminopropane) radical aldimine with deaza-Pyridoxal m
odel gtmp2 energy||0,2|N,0,1.81355347,-0.18206599,0.04377741|C,0,0.947
41936,0.77115116,0.03723614|H,0,1.28414324,1.81537706,0.04847353|C,0,-
0.48826363,0.53243319,0.01537358|C,0,-0.97434963,-0.80471483,0.0074753
7|C,0,-2.36060172,-1.07551797,-0.01167813|C,0,-2.76989292,1.33141867,-
0.01577192|C,0,-1.41017691,1.6148484,0.00355949|H,0,-3.48662746,2.1472
5093,-0.02530865|O,0,-0.14252611,-1.85689996,0.01843472|C,0,3.23777009
,0.14438738,0.05924021|C,0,3.8853654,-0.42790886,1.27984741|H,0,3.2919
8392,-0.70600973,2.14252471|H,0,3.35962826,1.24861833,0.06995989|H,0,4
.96700833,-0.47397353,1.34858707|C,0,3.89544506,-0.37521376,-1.2348151
3|H,0,0.79411191,-1.49496313,0.02990999|H,0,4.96799405,-0.15712447,-1.
23066638|H,0,3.76135299,-1.45700776,-1.32031909|H,0,3.44767501,0.10167
955,-2.1115294|C,0,-2.83453948,-2.50463125,-0.01896838|H,0,-2.45030092
,-3.04506613,-0.89095921|H,0,-2.47357481,-3.04624618,0.86217851|H,0,-3
.9265476,-2.55456757,-0.03347373|C,0,-0.94747436,3.05554713,0.01092515
|H,0,-0.35648266,3.2962687,0.90225188|H,0,-0.32983571,3.29664096,-0.86
203823|H,0,-1.80783822,3.72889377,-0.00196525|C,0,-3.23010929,0.006851
56,-0.02301658|H,0,-4.30090745,-0.18201408,-0.03798863||Version=IA32W-
G09RevD.01|State=2-A|HF=-593.1794512|MP2=-595.5469636|RMSD=1.795e-009|
PG=C01 [X(C12H16N1O1)]||@
